# Supplementary figures and images for: Inhibition of Phosphoglycerate Dehydrogenase Radiosensitizes Human Colorectal Cancer Cells under Hypoxic Conditions
Source: Cancers (Basel). 2022 Oct 15;14(20):5060. doi: 10.3390/cancers14205060 (PMC9599856; doi:10.3390/cancers14205060)

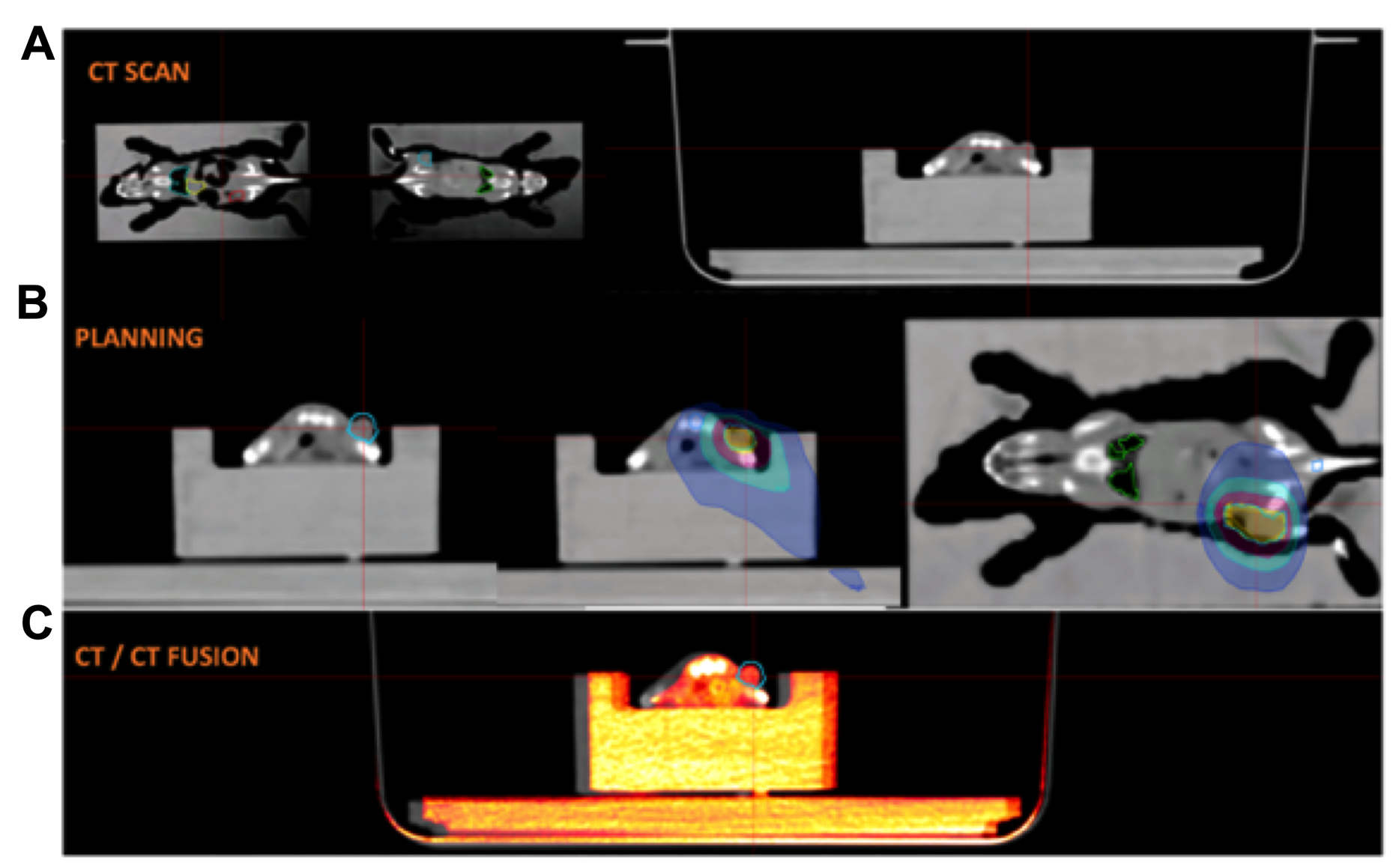

Supplement: Supplementary file 1 [file cancers-14-05060-s001.zip › Supplementary Figure S1 Delivery of radiation fractions to the tumors in HCT116 xenografts.tiff]

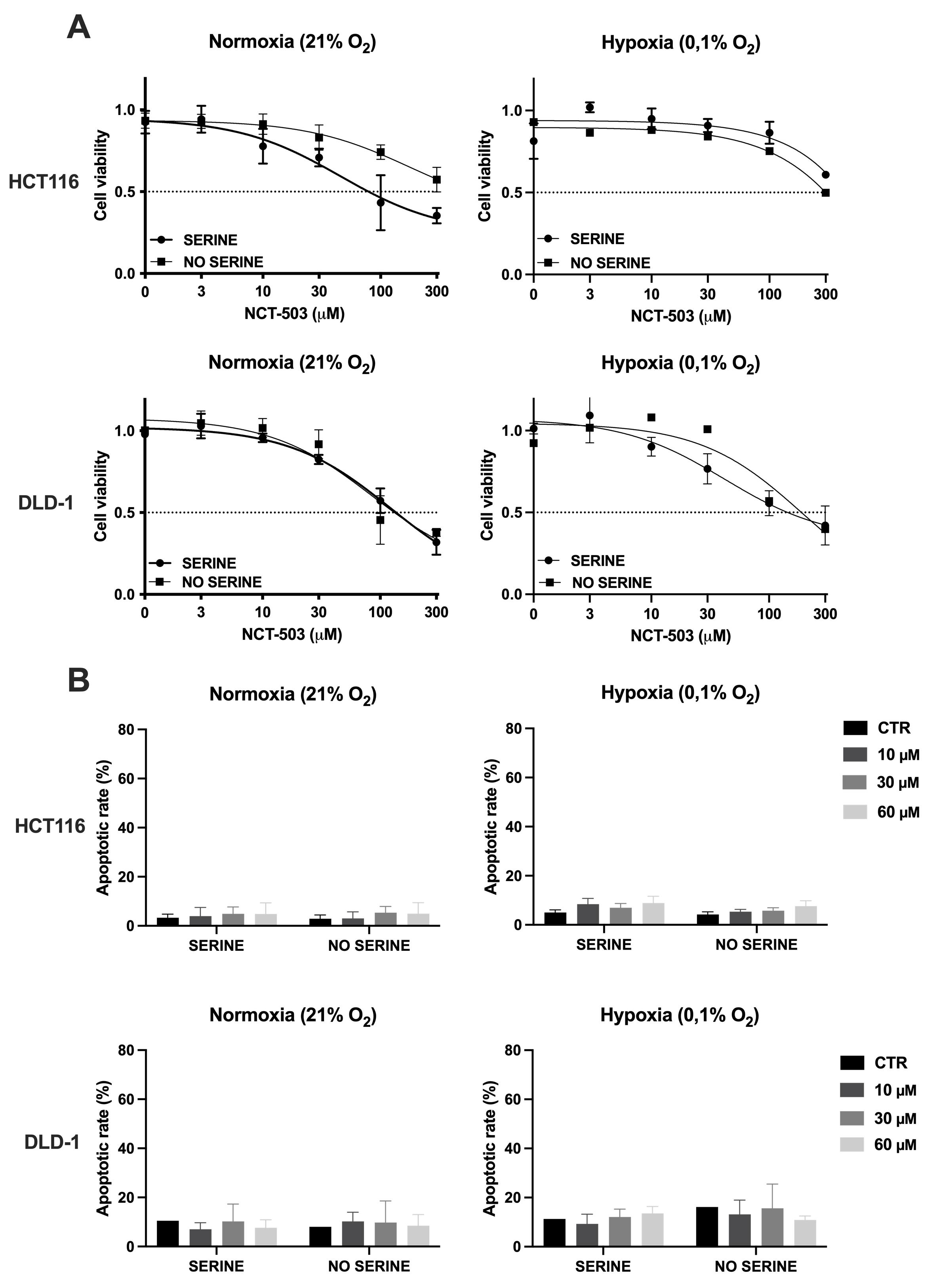

Supplement: Supplementary file 1 [file cancers-14-05060-s001.zip › Supplementary Figure S2 NCT-503 show a dose-dependent toxicity in human HCT116 and DLD-1 colorectal cancer cell lines.jpg]
